# Supplementary figures and images for: Detection and analysis of Serpin and RP26 specific antibodies for monitoring Schistosoma haematobium transmission
Source: PLoS Negl Trop Dis. 2025 Jan 24;19(1):e0012813. doi: 10.1371/journal.pntd.0012813 (PMC11759395; doi:10.1371/journal.pntd.0012813)

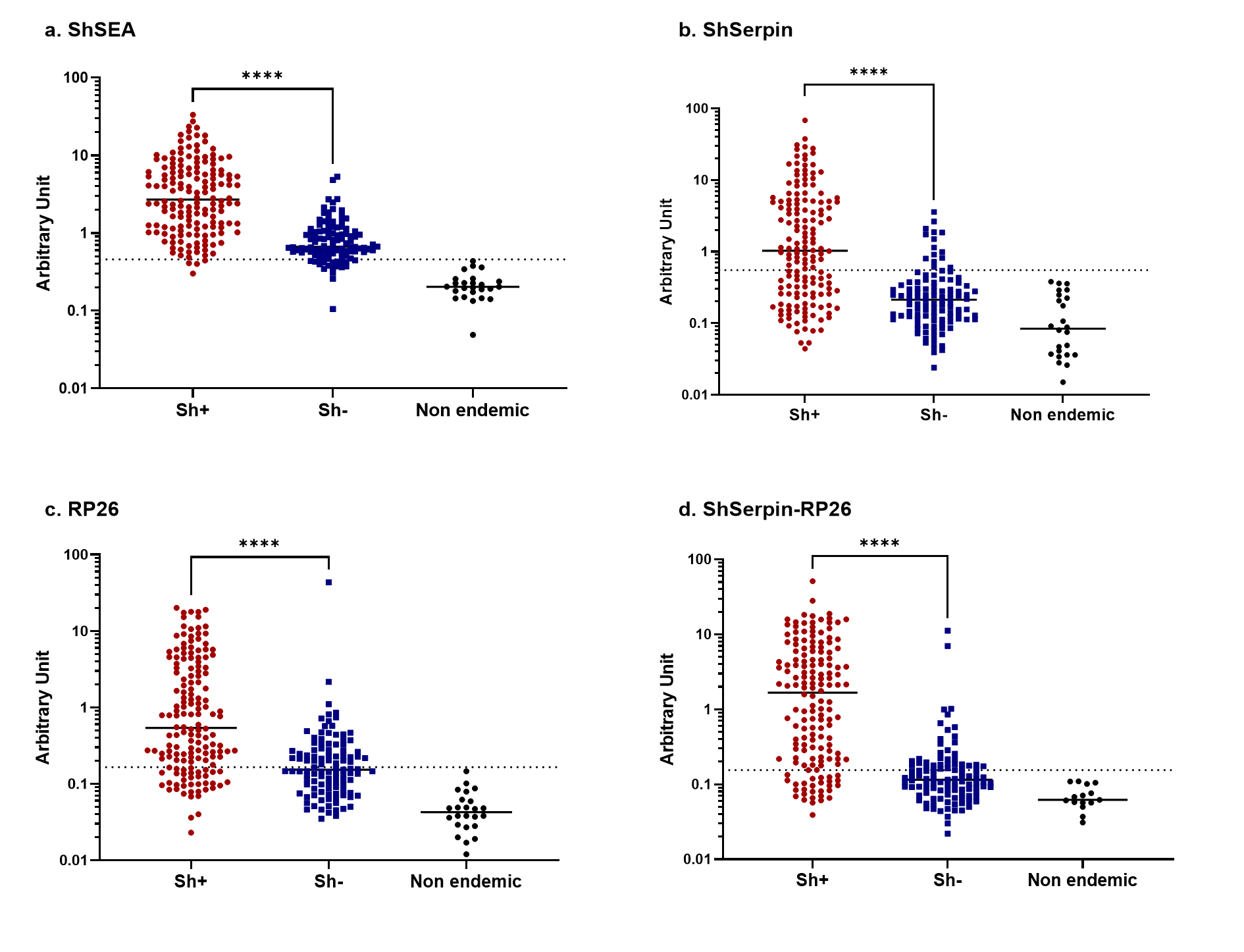

Supplement: S1 Fig — Total IgG levels against (a) ShSEA, (b) ShSerpin, (c) RP26, (d) ShSerpin-RP26 mixture were analyzed among S. haematobium infected (Sh+)/uninfected (Sh-) Kwale samples and non-endemic control samples from Japan (NC). pCAA500 indecisive results are considered as pCAA positive. The bars represent the median values of arbitrary units in each group. The dotted lines show the cut-off values determined by the geometric mean plus 3 SD of non-endemic controls (Japanese, n = 25)’ unit values. The cut-off value of each antigen was; 0.459 for ShSEA, 0.552 for ShSerpin, 0.165 for RP26, and 0.155 for ShSerpin-RP26 mixture. Statistical significance was set at p < 0.05 and is shown using asterisks: **** = p < 0.0001. Sh+, S. haematobium infection positive (pCAA500 positive and/or urine egg positive), shown in red dots (n = 160). Sh-, S. haematobium infection negative (pCAA500 negative and urine egg negative), shown in blue dots (n = 109). NC, non-endemic controls (Japanese), shown in black dots (n = 25). (TIF) [file pntd.0012813.s005.tif]

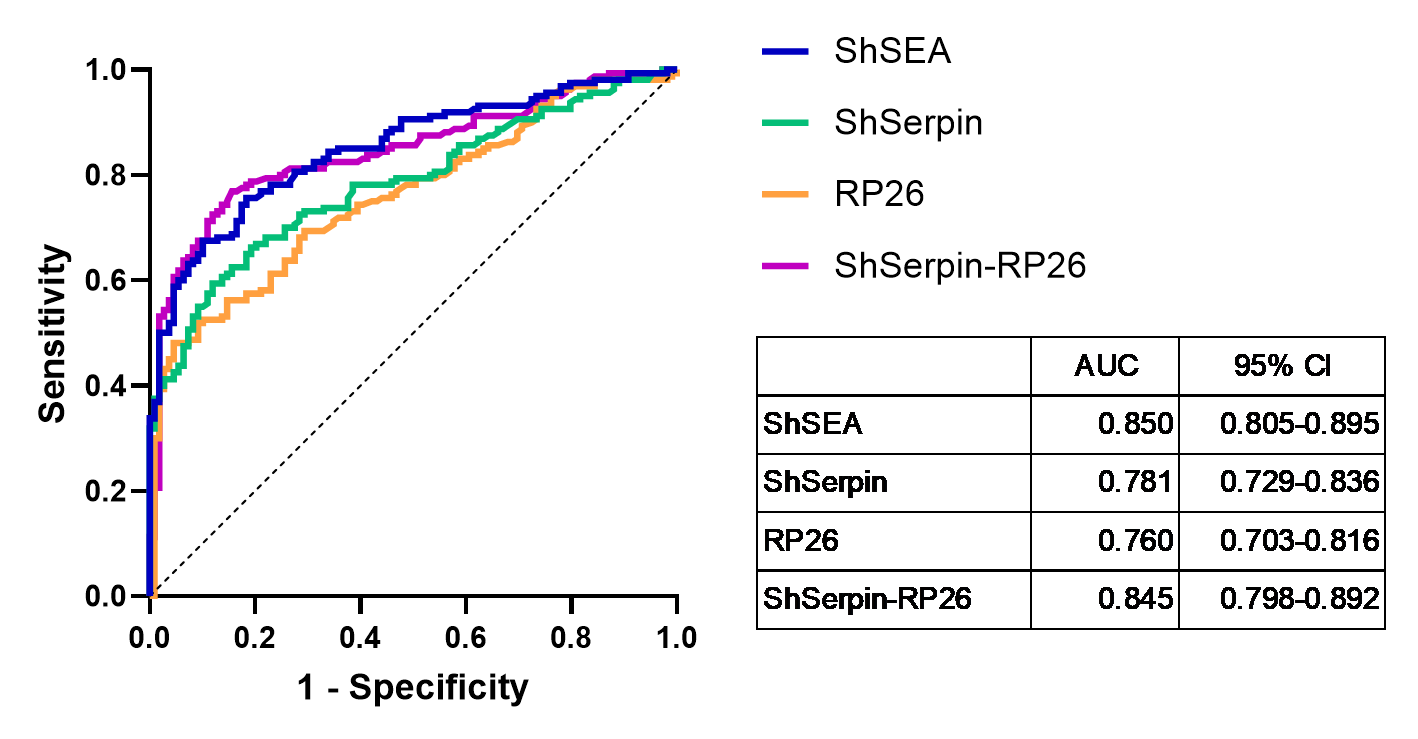

Supplement: S2 Fig — The ROC curves were generated from the ELISA results of Kwale samples infected (Sh+, n = 160) and uninfected (Sh-, n = 109) with S. haematobium. pCAA500 indecisive results were considered as CAA positive. The area under curve (AUC) of ShSEA, ShSerpin, RP26 and ShSerpin-RP26 mixture was 0.850, 0.783, 0.760 and 0.845, respectively. (TIF) [file pntd.0012813.s006.tif]

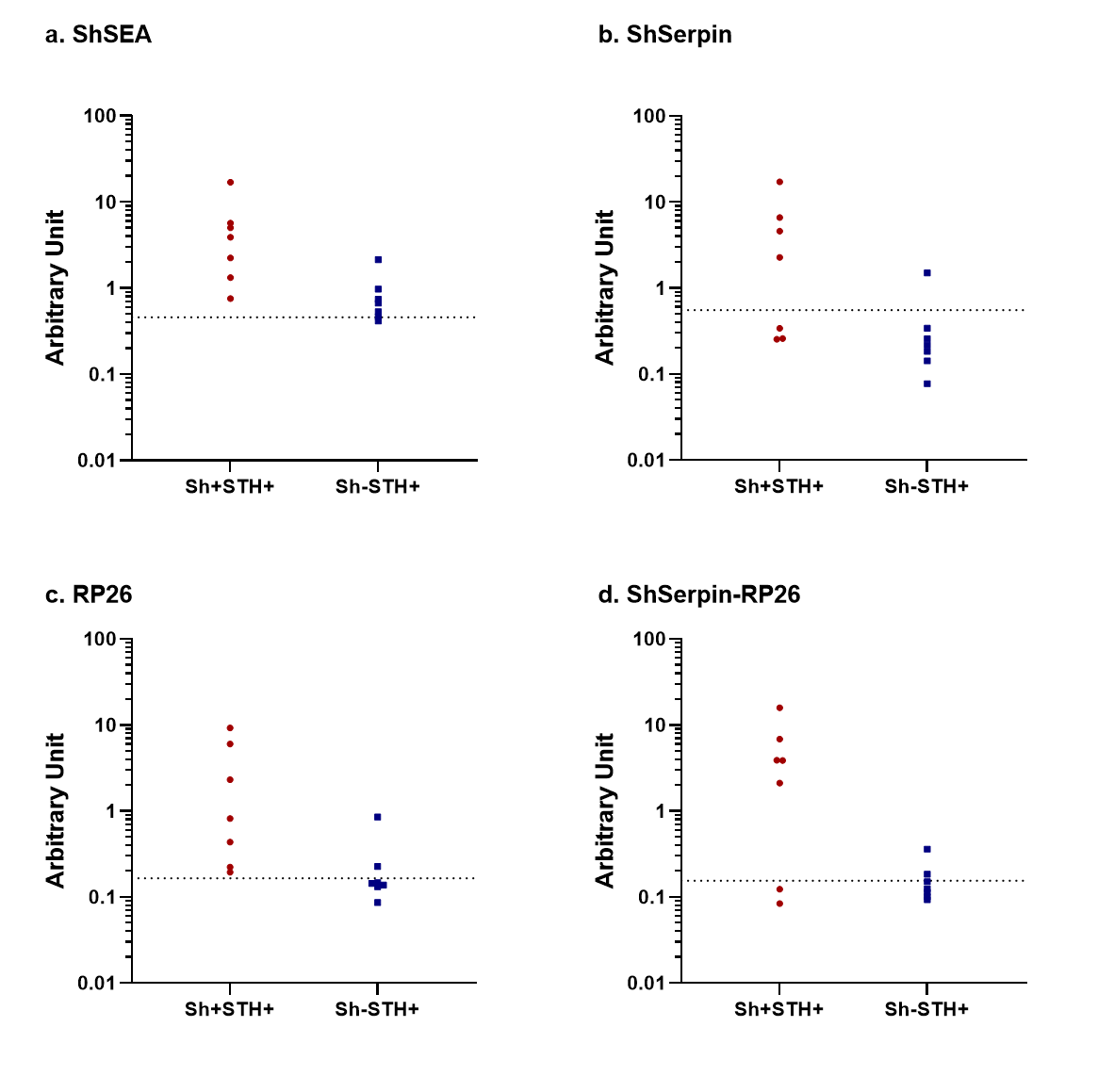

Supplement: S3 Fig — Total IgG levels against (a) ShSEA, (b) ShSerpin, (c) RP26, (d) ShSerpin-RP26 mixture were analyzed among STH infections positive plasma samples with or without S. haematobium infection from Kwale. The dotted lines show the cut-off values determined by the mean + 3SD of non-endemic controls’ units. Sh+STH+, S. haematobium infection positive (PCAA500 positive and/or urine egg positive) and STH infection positive (confirmed by four Kato-Katz slides), shown in red dots (n = 7). Sh-STH-, S. haematobium infection negative (PCAA500 negative and urine egg negative) and STH infection negative (confirmed by four Kato-Katz slides), shown in blue dots (n = 7). (TIF) [file pntd.0012813.s007.tif]
