# Supplementary material for: Detection and analysis of Serpin and RP26 specific antibodies for monitoring Schistosoma haematobium transmission
Source: PLoS Negl Trop Dis. 2025 Jan 24;19(1):e0012813. doi: 10.1371/journal.pntd.0012813 (PMC11759395; doi:10.1371/journal.pntd.0012813)
Supplement: Attachment — Submitted filename: PLOS NTD.docx [file pntd.0012813.s008.docx]

This is a good research towards developing diagnostics for elimination of schistosomiasis in the endemic region. The outline of the research is alright. However, I am not clear with concept of the assay in the following areas:

- what’s your analyte

- what’s your recognition element

- Are you using antigen to detect antibody or otherwise

- Where is the graphical abstract to clarify the layout of the biosensor. Provide the graphical abstract!

- Did you estimate the binding affinity? If no why?

- what’s the LOD or LOQ of the biosensor?

- line 416 schoolchildren should be separated . Check other errors

- Looks like your biosensor is ELISA based, and not for POC
