# Supplementary material for: Detection and analysis of Serpin and RP26 specific antibodies for monitoring Schistosoma haematobium transmission
Source: PLoS Negl Trop Dis. 2025 Jan 24;19(1):e0012813. doi: 10.1371/journal.pntd.0012813 (PMC11759395; doi:10.1371/journal.pntd.0012813)
Supplement: Attachment — Submitted filename: Response to Reviewers, PNTD-D-24-01409.docx [file pntd.0012813.s009.docx]

Response to Reviewers comments, PNTD-D-24-01409

Dear Reviewers,

We thank you for your careful review and helpful comments on the first version of our manuscript. Your input significantly improved our work. We have addressed all the concerns of the reviewers and modified the manuscript. We include point-by-point replies to the reviewer’s comments below. The original comments from the reviewers are shown in **bold**, and our replies are in standard type. We hope that our revised manuscript reflects all the suggestions from the reviewers and that our manuscript may now be considered suitable for publication in PLOS Neglected Tropical Diseases.

[Methods]

Reviewer #2

-**The method is clearly stated but I think it's it’s good to have the binding affinity, limit of detection data to validate the performance of the biosensor. I am not sure of the recognition element and antigen. There should be a graphical representation of the abstract.**
  We thank you for the suggestion. We have not tested the binding affinity because our target is not antigen but plasma IgG reactive to *Schistosoma haematobium*-derived recombinant antigens (ShSEA/ShSerpin/RP26) with various epitopes. The detection limit is also unavailable because it is impossible to prepare human anti-ShSEA/ShSerpin/RP26 IgG standards with known concentration. The graphical abstract of the antibody detection by ELISA using 96 well microplate is shown below:

Reviewer#3

- **Methods: What is the source of haematobium eggs to generate 'ShSEA'?**
  Thank you for the question. ShSEA used in this study was produced from *Schistosoma haematobium* eggs isolated from infected Golden Syrian hamster livers. The liver material is from the *S. haematobium* life cycle kept at Leiden University Medical Center since 1993 with a NAMRU-3, Cairo, Egypt strain. We added the description to the revised manuscript (now line 224-226).

[Results]

Reviewer #2

- **There is clarity in the presentation of results but binding affinity data will do more Justice to performance of the biosensor.**
  Thank you for the suggestion. We have not tested the binding affinity for the reasons stated above.

Reviewer #3

- **For ELISAs, how does the inclusion of the (+) plasma reference in the assay aid in your interpretation? Can the non-endemic control alone be used for analysis?**
  Thank you for the question. We used Sh+ plasma to define the arbitrary unit value to the measured absorbance values of each test sample by fitting the curves generated by standard positive plasma references (line 245-252, Method part). This allowed us to examine the correlation between PCAA500 values and antibody levels (The cut-off levels were set as the geometric mean plus 3 SD of non-endemic controls, and the determinations of positive/negative were based on this cut-off value).
- **Figure 5: Will it be helpful to know the value of the cutoff for each of the 4 assays?**
  Thank you for the suggestion. The cut-off values of the four antigen sets were 0.459 U for ShSEA, 0.552 U for ShSerpin, 0.165 U for RP26 and 0.155 U for the ShSerpin-RP26 mixture (we changed its description section from ‘Method’ part to the ‘Results’ part; now line 298-300). The cut-off values shown in Figure 5 are the same as those of other figures. We added each cut-off value description to all figure legends of Fig. 4 and Fig. 6 (Fig. 5 in the original manuscript).
- **Did the authors test different concentrations of the ELISA capture (1ug/mL); the ShSEA is an imperfect reagent, but will increasing capture conc. improve delineation for diagnosis of active infection?**
  We thank you for raising this point. We also tested different concentrations (such as 2 µg/mL or 0.5 µg/mL). As SEA contains numerous cross-reactive antigenic molecules, increasing coating antigen concentration causes higher cross-reactions. Thus, the current concentration (1 µg/mL) was optimal considering both sensitivity and specificity.

[Conclusions]

Reviewer#3

- **In conclusion, the limits of the assay are discussed, but the data is relevant to our field given the limitations of detection systems. A more conservative title may capture the conclusions more aptly, such as: 'Detection and analysis of Serpin and RP26 IgG for monitoring Schistosoma haematobium transmission'**
  Thank you for the suggestion. We have changed the title based on your input.

[Editorial and Data Presentation Modifications]

Reviewer#3

- **Minor edit in the References: #13 includes doi; #14 includes doi; has word duplication**
  Thank you, we have deleted the doi of #13 and #14 and corrected the duplication of ‘J Infect Dis.’ in #14.

[Summary and General Comments]

Reviewer#1

- **ELISAs using the ShSerpin-RP26 mixture as antigen cross-react with sera from patients infected with *S. mansoni*. Therefore, it should be mentioned in the discussion that caution should be exercised when using this ELISA in areas where schistosomiasis mansoni and schistosomiasis bilharzia are prevalent.**
  We thank you for your suggestion. We added the description in the discussion part (now line 516-519).
- **The ability to detect active infection is an important feature of the ELISA using the ShSerpin-RP26 mixture as antigen. Figure S-6 is a key element in demonstrating this and should be shown in the text rather than in the Supplement.**
  Thank you for the recommendation. We changed S6 Fig to Fig 5.

[Question and comments in the attached file]

- **what’s your analyte**
  Thank you for the comment. Our target is human plasma antibody (IgG), which reacts to *S. haematobium*-derived recombinant protein antigens (ShSerpin, RP26) /crude antigen (ShSEA).
- **what’s your recognition element**
  At this point, we do not know so far. We are going to determine it in the future study.
- **Are you using antigen to detect antibody or otherwise**
  We used recombinant protein antigens to detect antibodies (IgG) in human plasma samples by ELISA.
- **Where is the graphical abstract to clarify the layout of the biosensor. Provide the graphical abstract!**
  We used a widespread 96-well transparent plate to measure the absorbance. The graphical abstract is shown above.
- **Did you estimate the binding affinity? If no why?**
  No, we did not because we cannot estimate the binding affinity for the reasons stated above.
- **what’s the LOD or LOQ of the biosensor?**
  We cannot determine the LOD or LOQ, because our method is microplate ELISA detecting antibodies reacting to various epitopes on *S. haematobium*-derived antigens, and the antibodies cannot be quantified.
- **line 416 schoolchildren should be separated. Check other errors**
  Thank you for the suggestion. We changed it to “school children”.
- **Looks like your biosensor is ELISA based, and not for POC**
  Thank you for the comment. As you pointed out, our antibody detection method is currently ELISA-based. We think our antibody detection assay could be developed into a point-of-care lateral-flow-based assay in the future by capturing antibodies that react to the coated antigens, such as ShSerpin and RP26.
